# Synthesis and Microwave Absorption Properties of Sulfur-Free Expanded Graphite/Fe_3_O_4_ Composites

**DOI:** 10.3390/molecules25133044

**Published:** 2020-07-03

**Authors:** Jian Sun, Lijie Li, Rui Yu, Xianlong Ma, Shaohua Jin, Kun Chen, Shusen Chen, Xijuan Lv, Qinghai Shu

**Affiliations:** 1 Xi’an Modern Control Technology Research Institute, Xi’an 710065, China; 13671115763@163.com (J.S.); lilijie2003@bit.edu.cn (L.L.); xjyr203@sina.com (R.Y.); yhao@fiu.edu (X.M.); jinshaohua@bit.edu.cn (S.J.); k.chen@bit.edu.cn (K.C.); chenbit@126.com (S.C.); 2 School of Materials Science and Engineering, Beijing Institute of Technology, Beijing 100081, China

**Keywords:** expanded graphite, non-toxic, sulfur-free, Fe_3_O_4_, sandwich-like composite structure, electromagnetic attenuation

## Abstract

In this study, sulfur-free expanded graphite (EG) was obtained by using flake graphite as the raw material, and EG/Fe_3_O_4_ composites with excellent microwave absorption properties were prepared by a facile one-pot co-precipitation method. The structure and properties of as-prepared EG/Fe_3_O_4_ were investigated by scanning electron microscopy (SEM), transmission electron microscopy (TEM), Fourier transform infrared (FT-IR), X-ray diffraction (XRD), Raman, X-ray photoelectron spectrometry (XPS), thermogravimetric (TG), and vibrating sample magnetometry (VSM) characterizations. The Fe_3_O_4_ intercalated between the layers of expanded graphite forms a sandwich-like structure which is superparamagnetic and porous. When applied as a microwave absorber, the reflection loss (R_L_) of EG/Fe_3_O_4_ reaches −40.39 dB with a thickness of 3.0 mm (10 wt% loading), and the effective absorption bandwidth (EAB < −10 dB) with R_L_ exceeding −10 dB is 4.76–17.66 GHz with the absorber thickness of 1.5–4.0 mm. Considering its non-toxicity, easy operation, low cost, suitability for large-scale industrial production, and excellent microwave absorbing performance, EG/Fe_3_O_4_ is expected to be a promising candidate for industrialized electromagnetic absorbing materials.

## 1. Introduction

Electromagnetic wave absorbing (EMWA) materials with strong absorption, wide absorption bandwidth, thin thickness, light weight, and high thermal stability are widely used in military and civilian fields [1,2,3,4,5,6,7,8,9,10]. During the past decades, expanded graphite (EG) was widely researched in the area of EMWA considering its wider source, lower cost, larger surface area, high surface activity, rich pore structure, etc., which can be applied in lithium ion batteries [11], biomedicine [12], or electronic heat dissipation [13]. EG also has a wide range of applications in the field of electromagnetic shielding [1,14,15].

However, the preparation of EG by chemical oxidation is mainly carried out by using concentrated sulfuric acid; as a result, the residual sulfur is easy to cause corrosion of metals in applications, especially in high ambient temperature environments [15,16,17,18]. Therefore, the preparation of sulfur-free EG is extremely important for production applications. On the other hand, Fe_3_O_4_ has the advantages of superparamagnetism, high saturation magnetic strength, excellent biocompatibility, and excellent magnetic response [19,20]. It has a wide range of applications in new sensing materials [21], medicine [22], and catalysis [23]. At the same time, Fe_3_O_4_ is also a vital magnetic medium that can be used in electromagnetic absorbing materials [24,25,26,27,28,29,30,31,32,33,34,35].

Recently, numerous studies reported that graphene/Fe_3_O_4_ composites were successfully fabricated with excellent absorbing properties. Zeng et al. [26] synthesized Air@rGO (reduced graphene oxide)/Fe_3_O_4_ by a water-in-oil (W/O) emulsion technique followed by a calcination process. The minimum reflection loss (R_L_) value reaches −52 dB at 10 GHz with a thickness of 2.8 mm. Wang et al. [30] reported a simple hydrothermal method to synthesize Fe_3_O_4_@ZnO/rGO composites with a wide effective absorption bandwidth up to 12 GHz with reflection loss R_L_ < −10 dB and a minimal R_L_ (−34 dB) at 6.7 GHz. Huang et al. [32] Through ultrasonic and thermal reduction processed octahedral Fe_3_O_4_/rGO composites, the bandwidth of R_L_ exceeded −20 dB (99% absorption) over 5 GHz (12–16 and 17–18 GHz). Wu et al. [33], through a one-pot solvothermal method, synthesized Fe_3_O_4_/rGO, obtaining a minimum R_L_ of −22.7 dB at 3.13 GHz. Although graphene/Fe_3_O_4_ composites have been successfully fabricated, the traditional method requires at least two or three oxidation processes to synthesize graphene and the use of large quantity organic solvents to obtain the target composite. In addition, there are many other disadvantages, such as poor growth control of Fe_3_O_4_ and low yield of composite materials. Obviously, making a high-efficiency microwave-absorbing carbonaceous material in a one-step, non-toxic, cheap, and simple preparation process remains a huge challenge.

Herein, we report the preparation of expanded graphite by the method of mixing acid with nitric acid and phosphoric acid. The expansion rate was up to 300 mL/g which can be proved from Figure S1 in the Supporting Information. The sulfur-free EG intercalation Fe_3_O_4_ was synthesized through a facile one-pot co-precipitation method, which was free of additional processing, non-toxic, easy to operate, low in cost, and easy to scale. In addition, the particle size of Fe_3_O_4_ prepared by the mechanical stirring method was more uniform, and the particle size distribution was concentrated in the range of 10–20 nm; hence, the growth control of Fe_3_O_4_ was good, as shown in Figure S2 in the Supporting Information. The morphology, crystal structure and defects, thermal properties, and absorbing properties of the samples were investigated in detail, and the synthesized material showed effective EMWA absorption.

## 2. Experimental Section

### 2.1. Preparation of Sulfur-Free EG

EG was synthesized by chemical oxidation of natural flake graphite. In a typical procedure, 2.0 g graphite was continuously stirred with a combination of 5 mL nitric acid (68% concentration) and 15 mL phosphoric acid (85% concentration) solution under 45 °C. After this, 0.4 g potassium permanganate (KMnO_4_) was added into the solution when the temperature was raised to 45 °C, and the products was obtained by suction filtration after an 80 min intercalation process. The product was washed with deionized water until the pH was 7.0 and then dried at 60 °C. Finally, sulfur-free EG was obtained by sparking the production in a muffle furnace under 900 °C for 1 min.

### 2.2. Preparation of Sulfur-Free EG/Fe_3_O_4_ Composites

In order to prevent the oxidation of Fe^2+^ during the reaction, the molar ratio of Fe^3+^ to Fe^2+^ was set to 1:1. EG (100 mg) was weighed into a 100 mL beaker containing 35 mL of water and 10 mL of ethanol. Then, a certain amount of 2.5 mmol of Fe^2+^ and Fe^3+^ was directly added to the mixed solution, followed by ultrasonic dispersion for 30 min. The solution was then transferred into a 250 mL three-necked flask and another 30 min of mechanical stirring was done. When the temperature was raised to 80 °C, ammonia water was added dropwise until the solution pH was 11. Subsequently, the black products were washed several times with deionized water and ethanol and dried at 80 °C under vacuum and denoted as S1. For comparison, different molar masses of Fe^2+^ and Fe^3+^ (1.00 mmol, 0.75 mmol, 0.50 mmol, 0.25 mmol) being added to the reaction system and the corresponding products were labeled as S2, S3, S4, and S5, respectively. The yield of the composites was roughly obtained by the mass ratio of the actual composite to the theoretical one. Through calculation, we found that the yields were all about 90% according to the thermogravimetric (TG) test.

### 2.3. Characterization

The morphologies of samples S1–S5 were characterized by scanning electron microscopy (SEM; Hitachi S-4800, 10 kV) and transmission electron microscopy (TEM; FEIF20, 200 kV). X-ray diffraction (XRD) patterns were obtained with a Bruker D8 Advance X-ray diffractometer (XRD, Bruker D8 Advance, Cu Ka radiation, λ = 0.15406 nm, 8°/min). X-ray photoelectron spectrometry (XPS) was measured by K-Alpha Fourier transform infrared (FT-IR, Nicolet FTIR-170SX, 4000–500 cm^−1^, room temperature) absorption spectra, Raman (HORIBA Lab RAM HR Evolution, 500–2000 cm^−1^, 514 nm), thermogravimetric analysis (TGA; Shimadzu Corporation DTG-60, from 50 °C to 1050 °C in air). The magnetic properties of samples were characterized by a vibrating sample magnetometer (VSM, Riken Denshi, BHV-525) at room temperature. Electromagnetic (EM) parameters were measured by a vector network analyzer (NA, HP8720ES) over 2–18 GHz, in which powders were pressed to be toroidal samples (outer diameter: 7 mm, inner diameter: 3mm) according to the mass ratio 9:1 of paraffin to EG/Fe_3_O_4_ composite.

## 3. Results and Discussion

**Figure 1:**
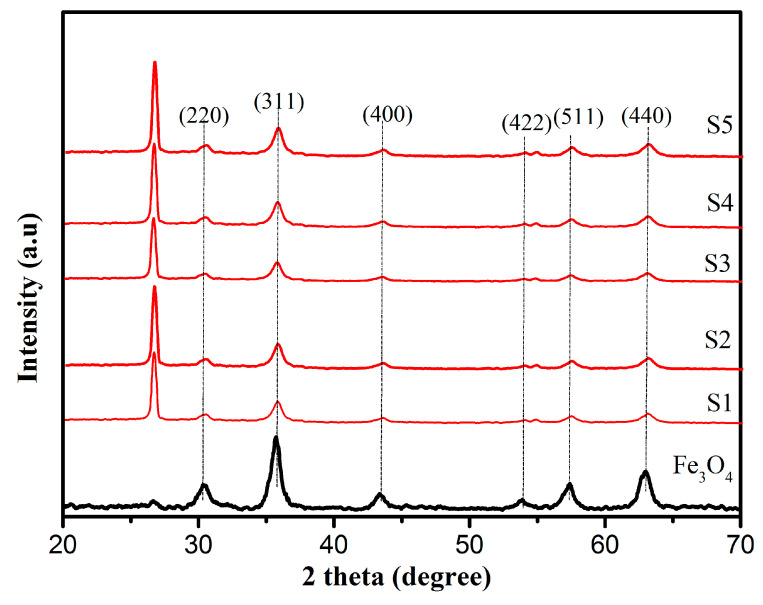
X-ray diffraction (XRD) patterns of Fe_3_O_4_, S1, S2, S3, S4, and S5.

XRD measurements were used to investigate the phase composition and the crystalline structure of the samples. Figure 1 shows the XRD spectra of Fe_3_O_4_ and EG/Fe_3_O_4_ composites. The diffraction peaks located at ~30.4°,~ 35.8°, ~43.4°, ~53.9°, ~57.9°, and ~63.1° can be perfectly indexed to the (220), (311), (400), (422), (511), and (440) planes of Fe_3_O_4_. As shown in Figure 1, the XRD patterns of EG show a sharp peak at 20~26.6°, corresponding to the graphitic structure of the short-range order in stacked graphene sheets [36]. No peaks corresponding to any other impurities were detected, suggesting that the EG/Fe_3_O_4_ composites were formed via the simplified co-precipitation method.

**Figure 2:**
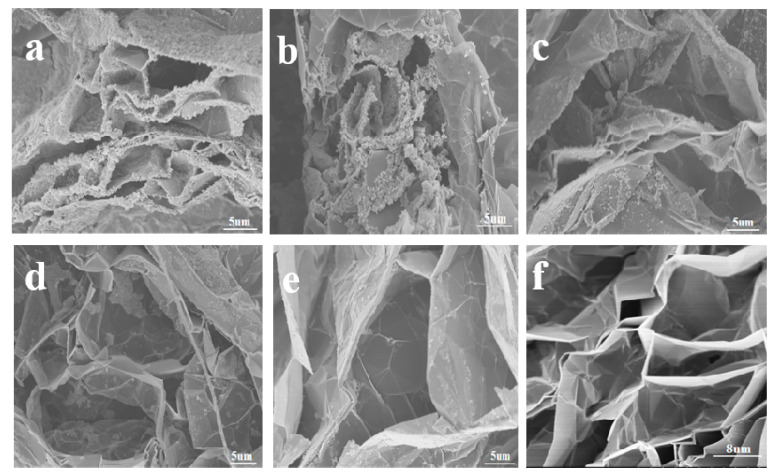
Scanning electron microscopy (SEM) images of expanded graphite (EG)/Fe_3_O_4_ prepared from different concentrations of Fe_3_O_4_ (gradually decreased from (**a**–**e**) in the figure) and EG (**f**), respectively.

With gradually decreased concentrations of Fe_3_O_4_ in EG (Figure 2a–e), it can be clearly seen that Fe_3_O_4_ was incorporated into the EG, forming a sandwich-like composite structure. Moreover, there were strong interfacial interactions between the Fe_3_O_4_ and EG, and most of the Fe_3_O_4_ was attached to the EG sheets (Figure 2a). This indicated the formation of the coordination bonds between Fe_3_O_4_ and EG, which is further demonstrated by IR characterization below. In addition, the generated large spaces between the layers of EG also favored multiple reflections to improve the microwave absorption performance.

**Figure 3:**
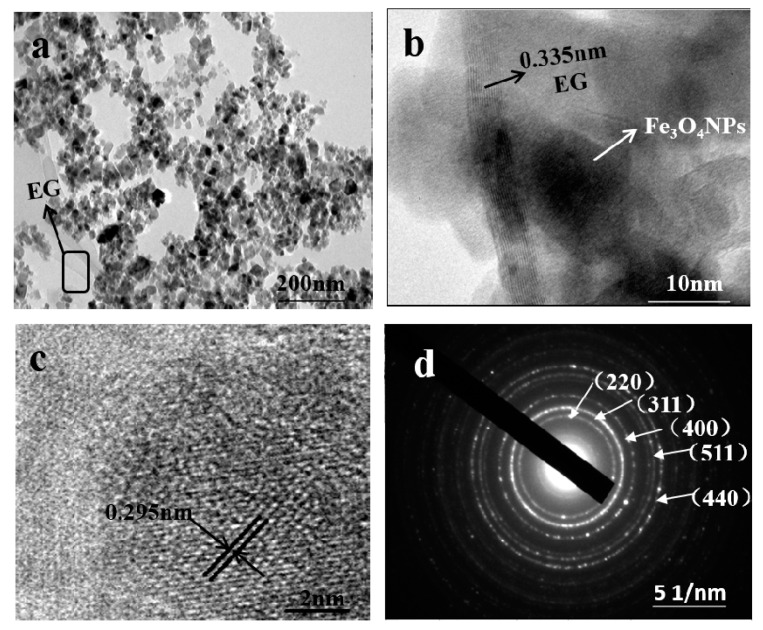
Transmission electron microscopy (TEM) images of EG/Fe_3_O_4_ (S1) composite (**a**); High Resolution Transmission Electron Microscope (HRTEM) image (**b**,**c**) and Selected Area Electron Diffraction (SAED) pattern (**d**) of EG/ Fe_3_O_4_ (S1) composite.

To get further insight into the nanostructure of samples, TEM of EG/Fe_3_O_4_ composite was carried out. As can be seen from Figure 3a, the Fe_3_O_4_ with an average size of 8–50 nm (can be proved from Figure S3 in the Supporting Information) is strongly attached to the EG sheets even after 30 min ultrasonic treatment with a frequency of 25 khz under room temperature, implying a strong interaction between the EG sheets and Fe_3_O_4_ [35], which could be attributed to a Fe-O bond, which was confirmed by FT-IR as below. In Figure 3b, black curved graphite sheets can be easily identified. The crystal plane spacing of 0.34 nm corresponds to the (002) crystal plane of EG. In Figure 3c, Fe_3_O_4_ has a lattice fringe of 0.295 nm interplanar spacing, corresponding to the cubic spinel crystal Fe_3_O_4_ (220) plane. The corresponding Selected Area Electron Diffraction (SAED) pattern as presented in Figure 3d is well indexed to the Fe_3_O_4_ structure with the (220), (311), (400), (511), and (440) plane, identifying the existence of Fe_3_O_4_, which is in accordance with the XRD results.

**Figure 4:**
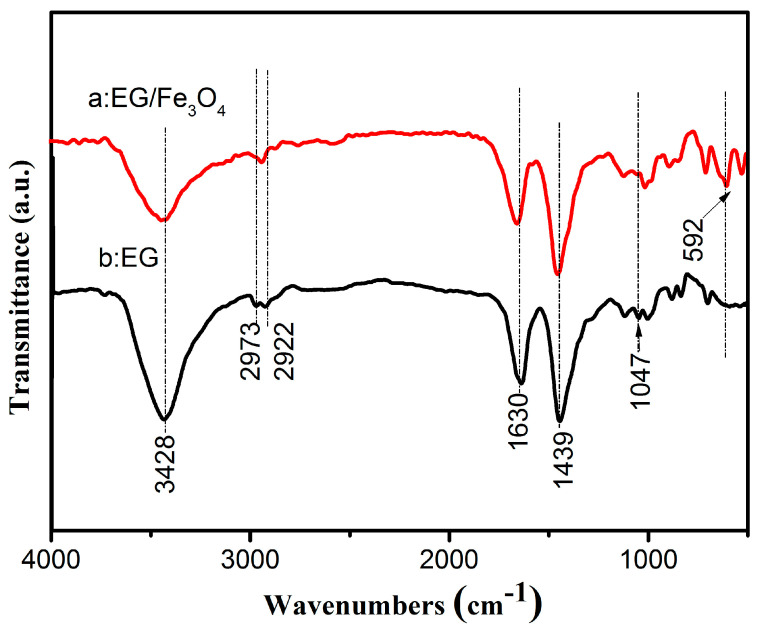
Fourier transform infrared (FT-IR) spectra of EG and EG/Fe_3_O_4_ composites.

The surface chemical environment of EG and EG/Fe_3_O_4_ composites was studied by FT-IR spectroscopy as presented in Figure 4. The intense absorption peaks positioned at ~3428 cm^−1^ can be ascribed to adsorbed H_2_O in sample S2. The peaks at ~1047, 1439, 1630, 2922, and 2973 cm^−1^ can be attributed to C-O stretching vibration of epoxide [30], tertiary C-OH groups stretching [31], C=C skeleton vibration, anti-symmetric stretching vibration of methylene, and stretching vibration absorption peak of a saturated C-H bond, respectively. Moreover, strong bands around 592 cm^−1^ in the samples can be ascribed to the stretching vibration of Fe-O bond of Fe_3_O_4_ [32], meaning that Fe_3_O_4_ was chemically bonded to the surface of EG through interaction with the Fe-O bond [33,34,35,37].

**Figure 5:**
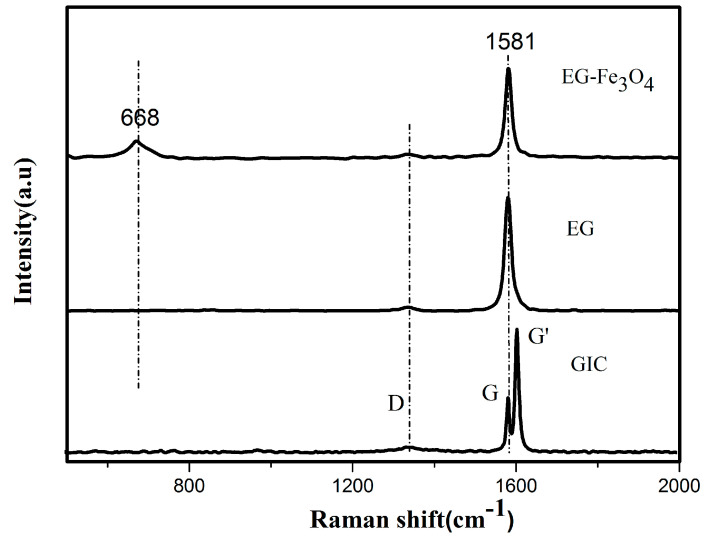
Raman spectra of graphite, graphene intercalation compounds (GIC), EG and EG/ Fe_3_O_4_ composite.

From the Raman spectrum of flake graphite, GIC (graphene intercalation compounds), EG, and EG/Fe_3_O_4_ in Figure 5, the sharp and strong absorption peak (G peak) at~1581 cm^−1^ is corresponding to the in-plane vibration of the sp2 carbon atom in graphite and the significant peak at 668 cm^−1^ in the spectrum of EG/Fe_3_O_4_ is corresponding to the characteristic peak of Fe_3_O_4_ [12].

Meanwhile, the small peak (D peak) at 1346 cm^−1^ and the G’ band at about 1620 cm^−1^ are associated with defects in the graphite sheet or graphite edge [38,39]. The D’ peak can be clearly seen in GIC, which is obviously higher than the G peak, indicating that the graphite sheet is opened by oxidation in the experiment to facilitate intercalation by the graphite sheet layer. The D peak on the spectrum of EG and EG/Fe_3_O_4_ is weak, indicating that the crystal structure of the EG is relatively intact and the degree of damage is low.

**Figure 6:**
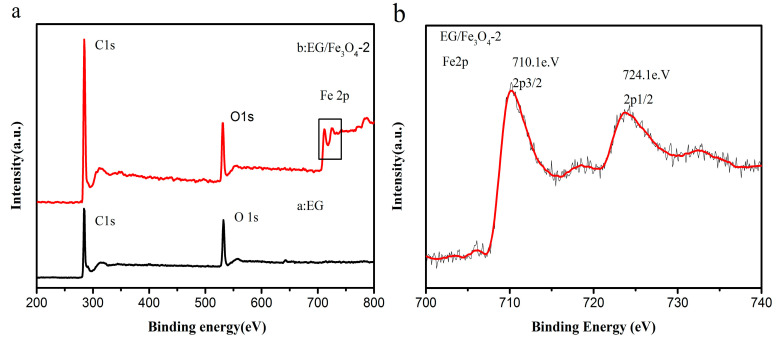
X-ray photoelectron spectroscopy (XPS): (**a**) wide scan, (**b**) Fe 2p spectrum of the EG/Fe_3_O_4_ composite.

X-ray photoelectron spectroscopy was used to determine the composition of the composites (Figure 6). The bands in the wide scan of S2 confirm the presence of C, O, and Fe elements. The observed of O 1s peak in GO at ~531.8 eV is shifted to a lower binding energy (530.6 eV) due to the attachment of the Fe_3_O_4_ in the EG/Fe_3_O_4_ composites [40]. In Figure 6b, the Fe 2p XPS spectrum exhibits two peaks at ~710.1 and ~724.1 eV, which can be assigned to the Fe _2p_ 3/2 and Fe _2p_ 1/2 spin-orbit peaks of Fe_3_O_4_, which is consistent with the reported values for Fe_3_O_4_ [17,41]. It can be seen that the left front is higher than the right peak, coinciding with the reported Fe_3_O_4_ [42], and there are no satellite peaks of iron oxides (such as α-Fe_2_O_3_ and γ-Fe_2_O_3_), which proves that there is no Fe_2_O_3_ phase in the hybrid.

**Figure 7:**
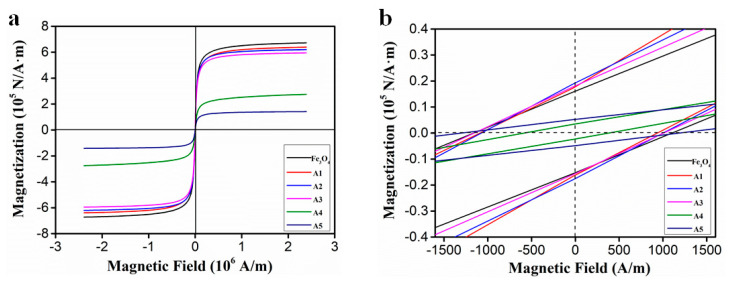
The magnetization curves of Fe_3_O_4_ and EG/ Fe_3_O_4_ (**a**) and an expanded view of the low-field magnetization curves (**b**).

As shown in Figure 7a, the magnetization curves of the six samples were measured at room temperature. It can be seen that the sample is superparamagnetic and has a small coercive force.

Table 1 reflects the specific magnetic parameters from Figure 7b, including the coercivity (*Hc*), remanent magnetization (*Mr*), and saturation magnetization (*Ms*). The *Ms* values decrease with a decrease of Fe_3_O_4_ loading amount, and the *Ms* value of the five samples is lower than that of the pure Fe_3_O_4_, which can be attributed to the influence of the nano-sized Fe_3_O_4_ and the presence of EG [25].

**Figure 8:**
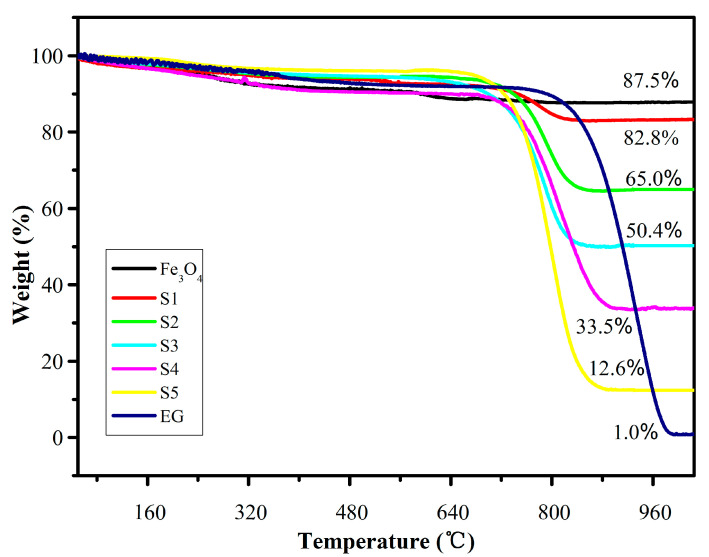
Thermogravimetric (TG) curves of Fe_3_O_4_ and composites S1–S5 under air atmosphere.

From the TG analysis curve, as shown in Figure 8, it can be seen that there are three steps between 0 and 1030 °C for the EG/Fe_3_O_4_ composites. Before the temperature rises to 100 °C, a slight weight loss occurs on the curve, which may be caused by the loss of the surface hydroxyl groups or adsorbed water. When the temperature is further increased, the product undergoes a relatively gentle decrease between 100 and 700 °C due to the destruction of the oxygen-containing functional group which is unstable on the surface of the EG, the destruction of the amino group on the surface of Fe_3_O_4_, and the evaporation of water vapor. CO_2_ gas is generated by the combustion of EG between 700 and 850 °C, resulting in a severe weight loss. After 850°C, a mostly steady value is reached corresponding to the mass of Fe_2_O_3_. Zong et al. [29,33] found that the oxidation and decomposition of graphene occurred between 350 °C and 510 °C. The EG/Fe_3_O_4_ complex prepared by the experiment has better heat resistance than the Reduced Graphene Oxide (RGO)/Fe_3_O_4_ complex.

To investigate the microwave absorption properties and mechanism of EG/Fe_3_O_4_, the electromagnetic parameters (relative complex permittivity, ε_r_ = ε’ − jε″, and relative complex permeability, μ_r_ = μ’ − jμ″) in the range of 2–18 GHz were measured. As is well known, the real permittivity (ε’) and real permeability (μ’) are correlated to the storage ability of electric and magnetic energy, while the imaginary permittivity ε″ and imaginary permeability μ″ symbolize the dissipation of electric and magnetic energy.

**Figure 9:**
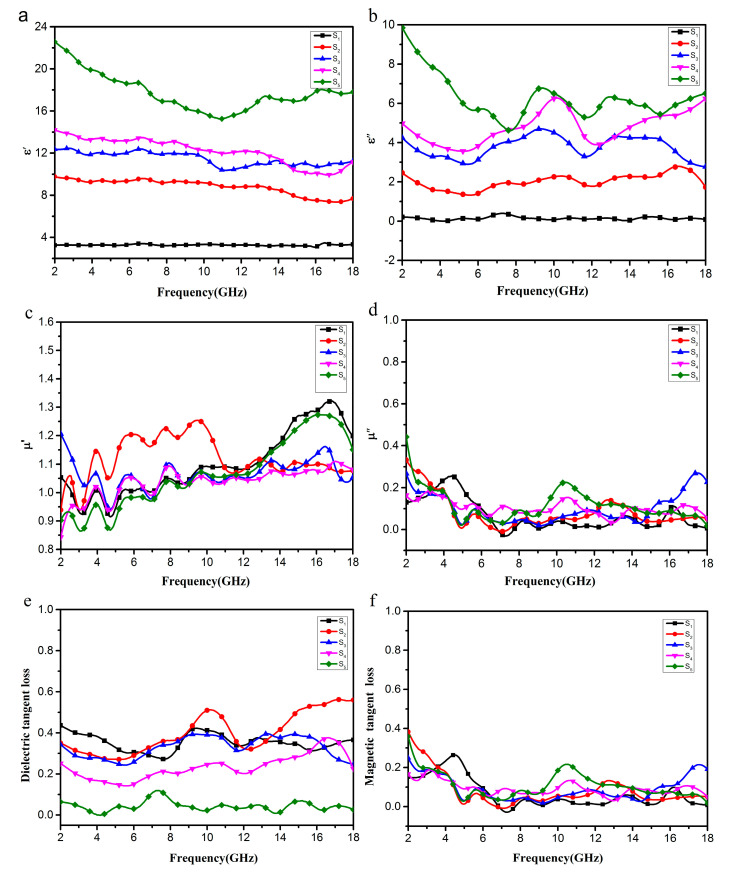
Frequency dependence on real parts (**a**) and imaginary parts (**b**) of the complex permittivity, real parts (**c**) and imaginary parts (**d**) of the complex permeability, and the corresponding dielectric loss tangents (**e**) and magnetic loss tangents (**f**) of composites S1–S5.

As shown in Figure 9a, the values of samples S5, S4, S3, and S2 in the 2–18 GHz range were 15.23–22.58, 9.98–14.22, 10.88–12.20, and 7.28–9.77, respectively, far higher than those of S1 with a high Fe_3_O_4_ content which were 2.99–3.31. The values of samples S2–S5 generally decrease with increasing frequency, but the values of the S1 samples are essentially unchanged, demonstrating a frequency-dependent dielectric response [4,29]. Meanwhile, as shown in Figure 9b, the values of ε″ are in ranges of 4.73–9.85, 3.16–6.21, 2.91–4.56, and 1.22–2.71, respectively. Although the values of these four samples fluctuated in the range of 2–18 GHz, they were still much higher than those of sample S1 which were in the range of 0.45–0.18 GHz. It is concluded that the samples with higher EG/Fe_3_O_4_ ratios show higher values of ε’ and ε″, which is unrelated to higher conductivities. This is because more EG sheets increase the electric polarization and electric conductivity, since ε’ is an expression of the polarizability of a material at microwave frequencies which consists of dipolar polarization and electric polarization [19,31]. As shown in Figure 9c,d, as for samples S1, S2, S3, S4, and S5, the μ’ and μ″ remain around 1 and 0 in the whole frequency range, respectively.

Based on the permeability and permittivity of samples, both the dielectric tangent loss (tan δ = ε″/ε’) and the magnetic tangent loss (tan δ_M_ = μ″/μ’) of the EG/Fe_3_O_4_ composites can be obtained. The values of tan δ are larger than 0.2 at almost 2–18 GHz for S1, S2, S3, and S4 as shown in Figure 9e, indicating the dielectric loss occurs at the entire frequency range. These results suggest that the EG/Fe_3_O_4_ composite has distinct dielectric loss properties. As for S1, the content of Fe_3_O_4_ in this sample is the highest, which is almost equal to the pure Fe_3_O_4_; hence, tan δ_M_ is the highest in these five samples. The resonance peak at about 10.2 GHz for the EG/Fe_3_O_4_ is possibly associated with the interfacial interaction between Fe_3_O_4_ and EG [26,27].

Based on the transmit-line theory, the reflection loss (R_L_) can be calculated by the following equations [25].

(1) $$Z_{in}=Z_{o}\sqrt{\frac{\mu_{r}}{\varepsilon_{r}}}\tanh(j\frac{2\pi fd}{c}\sqrt{\mu_{r}\varepsilon_{r}})$$

(2) $$R_{L}=20\log\left|\frac{Z_{in}-Z_{0}}{Z_{in}+Z_{0}}\right|$$

where Z_in_ is the input impedance of the absorber, μ_r_ and ε_r_ are respectively the relative complex permeability and permittivity, f is the frequency of microwaves, d is the thickness of the absorber, and c is the velocity of light in free space [43].

**Figure 10:**
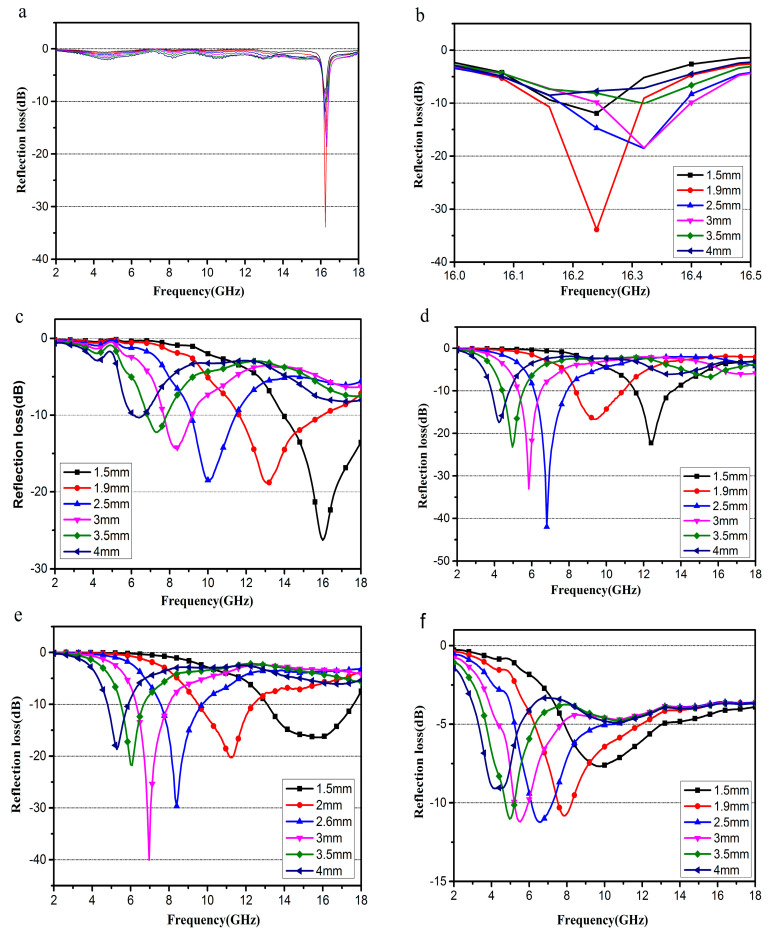
The calculated R_L_ for S1 (**a**,**b**), S2 (**c**), S3 (**d**), S4 (**e**) and S5 (**f**) with different thicknesses in the frequency range of 2–18 GHz.

As shown in Figure 10a, when the content of Fe_3_O_4_ is excessive, the R_L_ absorption peak of sample S1 only appears in the range of 16–16.5 GHz and is relatively sharp, and the range of the absorption peak movement is small with increasing thickness. When the sample thickness is 1.9 mm, the maximum R_L_ absorption can reach at −34.12 dB. The maximum R_L_ reaches −26.9 dB at 16.0 GHz, for S2 composite (Figure 10c) with a thickness of only 1.5 mm. In addition, a bandwidth of R_L_ less than −10 dB (90% absorption) can reach more than 4.1 GHz (from 13.9 to 18.0 GHz). For the composite S3 (Figure 10d), the maximum R_L_ reaches −36.8 dB at 7.7 GHz with a thickness of 2.5 mm, and a bandwidth of R_L_ less than −10 dB can reach 2.0 GHz. As for S4, Figure 10e shows that the maximum R_L_ reaches −40.39 dB at 6.9 GHz with a thickness of 3.0 mm, and EAB: <−10 dB with R_L_ exceeding −10 dB is 4.76–17.66 GHz with an absorber thickness of 1.5–4.0 mm.

It could be found that the R_L_ absorption peak moves towards the low-frequency direction with the increase of thickness, while the R_L_ absorption peak of the sample expands in the low-frequency direction with a decreasing content of Fe_3_O_4_. 

The excellent microwave absorbing performance of the EG/Fe_3_O_4_ nanoparticle composite is mainly attributed to two key factors: impedance matching and electromagnetic wave attenuation [19]. On one hand, the introduction of Fe_3_O_4_ has lowered the ε_r_ of the EG, and improved the equality of ε_r_ and μ_r_*,* which helps to improve the level of impedance matching [31]. On the other hand, the introduction of Fe_3_O_4_ could form more interfaces. The interfaces can act as polarization centers, which are favorable for microwave absorbing. Besides, the enormous aspect ratio, layered structure, and the existence of residual defects and groups of EG/Fe_3_O_4_ could cause multiple reflections, which could further enhance the microwave absorption ability of the composite [19,25].

## 4. Conclusions

In summary, EG/Fe_3_O_4_ composites with enhanced microwave absorption properties were prepared by a facile one-pot co-precipitation route, which avoided the usage of any additional chemical agents and inert gas. The preparation process of EG does not contain sulfur, which protects the environment and is easy to operate, low in cost, and easy to scale. In addition, the growth of composite materials could be well controlled by adjusting the molar masses of Fe^2+^ and Fe^3+^, and the yield is relatively high. The prepared EG/Fe_3_O_4_ sample does not only have better thermal performance than the widely studied RGO/Fe_3_O_4_ but also excellent absorbing properties. For the EG/Fe_3_O_4_(S4) composite, the maximum R_L_ reached −40.39 dB at 6.9 GHz with a thickness of 3.0 mm, and EAB: <−10 dB with R_L_ exceeding −10 dB is 4.76–17.66 GHz with an absorber thickness of 1.5–4.0 mm. It is believed that such sandwich-like structure composite will find applications in the microwave absorbing area as the base material of smoke bombs for electromagnetic interference, which could play a great role in electromagnetic shielding and heat dissipation.

## Figures and Tables

**Table 1:** Magnetic parameters of Fe_3_O_4_ nanoparticles and EG/Fe_3_O_4_ composites.

Samples	Hc (A/m)	Mr (10^4^ N/A.m)	Ms (10^5^ N/A.m)
Fe_3_O_4_	1152.61	1.62	6.72
S1	1076.19	1.93	6.39
S2	1049.92	1.68	6.20
S3	1150.22	1.78	5.95
S4	569.14	0.32	2.75
S5	1276.00	0.53	1.42

## Supplementary Materials

The following are available online. SEM images of NG, GIC, EG, and Fe_3_O_4_; The particle size distribution of the Fe_3_O_4_; The picture of 0.50 g EG; Black EG/Fe_3_O_4_ composite is rapidly separated under external magnetic field.
