# Supplementary material for: Synthesis and Microwave Absorption Properties of Sulfur-Free Expanded Graphite/Fe3O4 Composites
Source: Molecules. 2020 Jul 3;25(13):3044. doi: 10.3390/molecules25133044 (PMC7411745; doi:10.3390/molecules25133044)
Supplement: Supplementary file 1 [file molecules-25-03044-s001.pdf]

molecules

Supplementary Material

# Synthesis and Microwave Absorption Properties of Sulfur-Free Expanded Graphite/$Fe_3O_4$ Composites

**Jian Sun, Lijie Li, Rui Yu, Xianlong Ma, Shaohua Jin, Kun Chen, Shusen Chen, Xijuan Lv* and Qinghai Shu***

School of Materials Science and Engineering, Beijing Institute of Technology, Beijing 100081

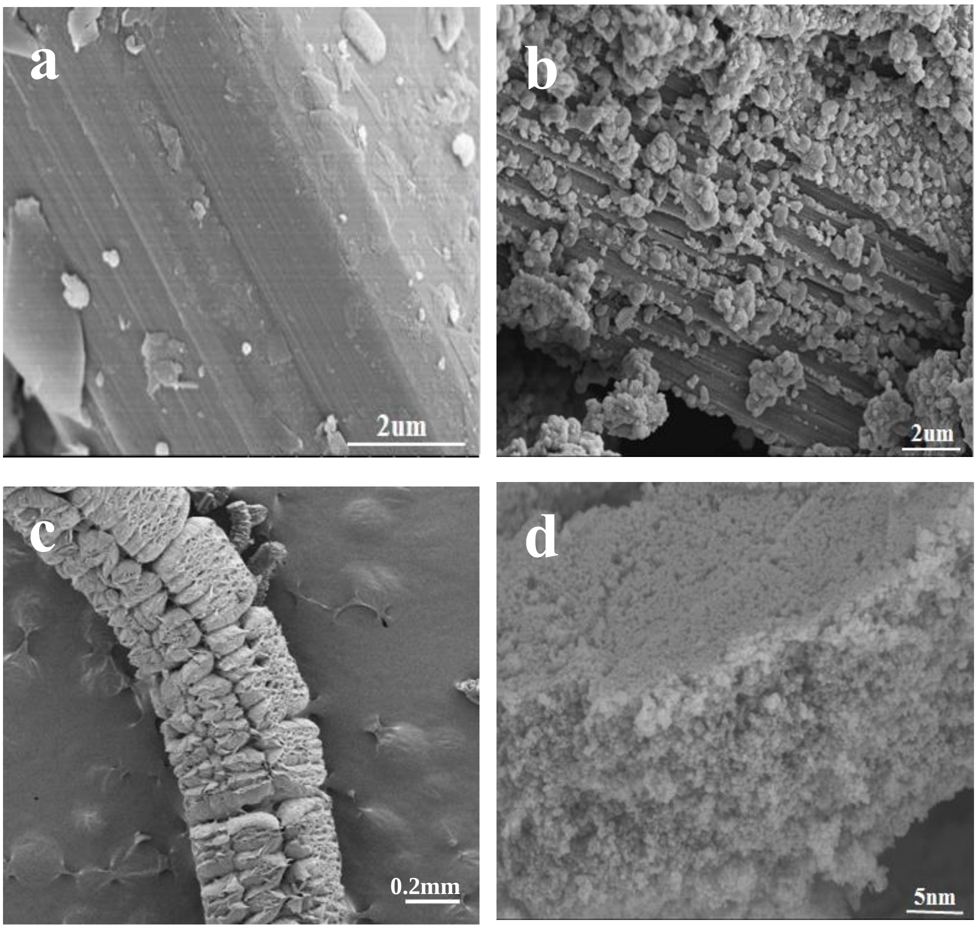


**Figure 1.** SEM images of NG (a), GIC (b), EG (c) and $Fe_3O_4$ (d).

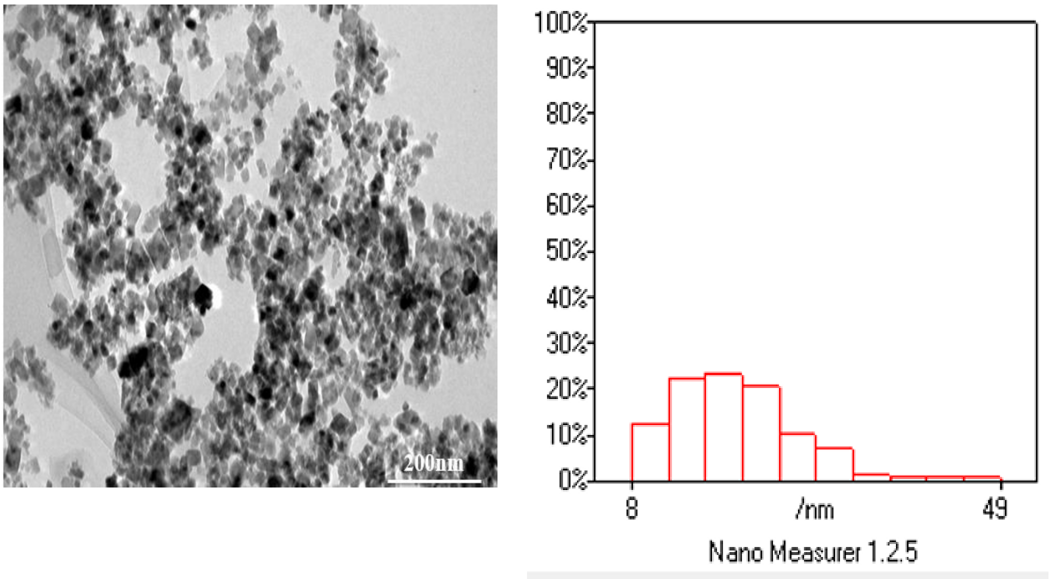


**Figure 2.** The particle size distribution of the $Fe_3O_4$

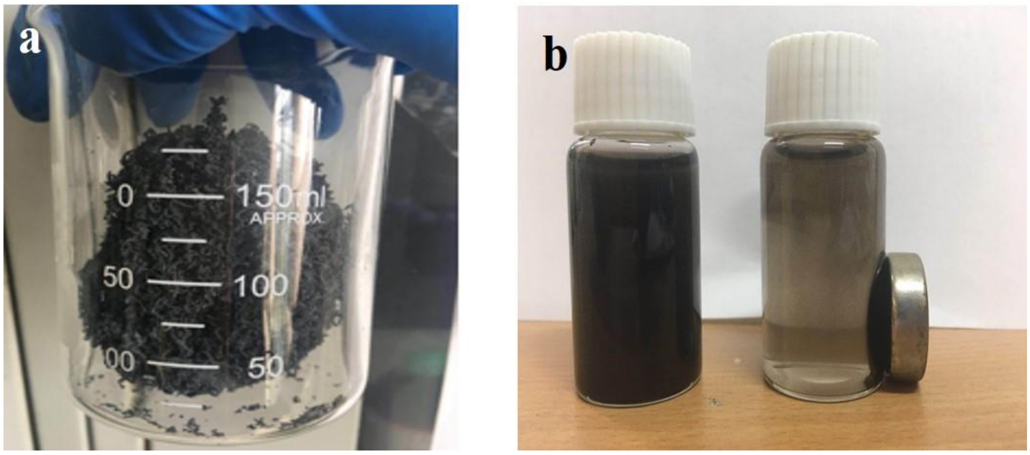


**Figure 3.** The picture of 0.5 g EG (a); Black EG/$Fe_3O_4$ composite is rapidly separated under external magnetic field (b).
